# Inactivation of Classical Swine Fever Virus in Porcine Serum Samples Intended for Antibody Detection

**DOI:** 10.3390/pathogens8040286

**Published:** 2019-12-05

**Authors:** Denise Meyer, Anja Petrov, Paul Becher

**Affiliations:** EU and OIE Reference Laboratory for Classical Swine Fever, Institute of Virology, Department of Infectious Diseases, University of Veterinary Medicine Hannover, Foundation, 30559 Hannover, Germany

**Keywords:** classical swine fever, pestivirus, *Flaviviridae*, virus inactivation, complement inactivation, detergent, Tween_20_, antibody detection, safety, sample transport

## Abstract

Shipping of serum samples that were taken from pigs infected with classical swine fever (CSF) virus is frequently requested with the objective of serological analyses, not only for diagnostic purposes but also for exchange of reference materials that are used as control material of diagnostic assays. On the basis of the fact that an outbreak with CSF is associated with enormous economic losses, biological safety during the exchange of reference material is of great importance. The present study aimed to establish a pragmatic approach for reliable CSF virus (CSFV) inactivation in serum without impairing antibody detection. Considering the fact that complement inactivation through heating is routinely applied, the basic idea was to combine heat treatment with the dilution of serum in a detergent containing buffer in order to facilitate the inactivation process. The results show that treatment of serum samples with phosphate buffered saline-Tween_20_ (final concentration = 0.15%) along with incubation at 56 °C for 30 min inactivated CSFV and such treatment with ≤ 0.25% PBS-Tween_20_ does not impair subsequent antibody detection by ELISA or virus neutralization test. This minimizes the risk of virus contamination and represents a valuable contribution to a safer CSF diagnosis on a national and international level.

## 1. Introduction

Classical swine fever (CSF) is one of the most important diseases in domestic pigs and wild boar and is notifiable to the World Organization of Animal Health (OIE). The disease is caused by the pestivirus classical swine fever virus (CSFV) which belongs to the virus family *Flaviviridae* [1]. An outbreak with CSF is associated with enormous economic losses due to the high mortality of the disease itself, as well as due to the widely conducted stamping out policy, which implies large-scale preventive culling of pigs along with transport and trade restrictions. Within the European Union, the last sporadic outbreaks of CSF occurred in Lithuania in 2009 and 2011, as well as in Latvia in 2012 up to 2015 [2]. However, CSF is still endemic in the feral and domestic pig population in many countries of Asia, Africa, South America, and the Caribbean. The recent emergence of CSF in Japan after 26 years absence provides an illustrative example for the continuous risk of reintroduction of the disease in CSF-free countries [3,4]. Maintenance of solid knowledge of the clinical disease and a rapid diagnosis of a CSF outbreak represent indispensable prerequisites for effective control of CSF. To keep diagnostic protocols up to date, constant verification and validation of the diagnostic methods is of great importance and includes the exchange of reference material for testing. In addition, the participation of the national reference laboratories in inter-laboratory comparison tests is obligatory, which implies the shipment of infectious sample material for testing. Not all national reference laboratories have the permission to work with infectious viruses. Therefore, those laboratories have to designate another official laboratory to perform diagnostic analysis of virus positive sample material. Furthermore, some institutions have a strict separation between serological (virus-free) and virological working departments. Laboratories or working departments that are not allowed to work with infectious sample material can receive samples that are tested negative for CSFV genome. In general, such samples are taken from animals at a late time point after infection and exhibit a high antibody concentration. However, these samples have been obtained from pigs that were infected with CSFV and despite appropriate measures and testing of this material, residual virus contents or contaminations cannot be ruled out in general. In order to minimize the risk of virus contamination at the consignee’s premises, especially for those laboratories that are not allowed to work with infectious virus, and to comply with legal shipping regulations, the present study aimed to establish a pragmatic approach for reliable CSFV inactivation in serum samples. Usually, complement inactivation (incubation at 56 °C for 30 min) of serum samples is performed prior to diagnostic analysis. However, this treatment results in a reduced viral titer of only about one log_10_ and is consequently insufficient to inactivate CSFV in serum samples because of virus protecting abilities of porcine serum at 56 °C and ambient temperatures [5]. For virus inactivation by heating, higher temperatures would be required. According to the European Commission (EC) legislation 2002/99/EC heating temperatures of 70 °C are necessary for elimination of infectious CSFV. However, due to denaturation of antibodies at such high temperatures, incubation of serum samples at 70 °C would severely impair the detection of antibodies in these samples. Previous studies have shown that a combination of heat treatment and the addition of the detergent Tween_20_ to serum samples results in inactivation of enveloped viruses and that these samples can be used for further serological analysis by antibody ELISAs without impairing the qualitative results. For Rift Valley fever virus as well as for Ebola virus (species *Zaire ebolavirus*), an incubation of the serum samples at 56 °C for 1 h in the presence of 0.5% Tween_20_ (*v*/*v*) is sufficient for virus inactivation [6,7,8]. A lower concentration of Tween_20_ (0.05%) resulted in inactivation of West Nile virus after an incubation at 56 °C for 1 h [9].

Considering the fact that complementary inactivation through heating (56 °C for 30 min) is routinely applied for serum samples prior to CSFV diagnosis, the basic concept of this study was to combine this heat treatment with the dilution of the serum sample in a detergent containing buffer [phosphate buffered saline(PBS)-Tween_20_] in order to provide a method for virus inactivation in serum samples without impairing the detection of antibodies against CSFV by diagnostic assays described within the EU legislation [10].

## 2. Results

### 2.1. Effect of Tween_20_ Concentration on Inactivation of CSFV in Serum Samples and Infectious Cell Culture Supernatants

**Figure 1:**
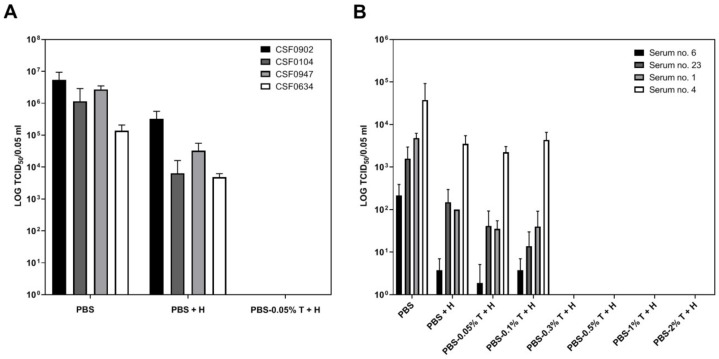
Classical swine fever (CSF) virus titer after sample treatment performed for virus inactivation. (**A**) Four infectious cell culture supernatants (two highly virulent strains, CSF0947 and CSF0634, as well as two moderately virulent strains, CSF0902 and CSF0104) and (**B**) four infectious serum samples were used for the experiment. The samples were 1:2 diluted in phosphate buffered saline (PBS)-Tween_20_ and incubated at 56 °C for 30 min. Two PBS controls were included; for the first, the samples were 1:2 diluted in PBS and incubated at 56 °C for 30 min (PBS + heat (H) control), whereas the second control was not treated with heat (PBS control). The four serum samples were treated with different Tween_20_ concentrations (0.05%, 0.1%, 0.3%, 0.5%, 1%, and 2%), whereas one concentration (0.05%) was used for the inactivation of infectious cell culture supernatants. Immediately after treatment, the samples were titrated. The experiment was performed in three independent runs. H = heat treatment at 56 °C for 30 min; T = Tween_20_.

**Figure 2:**
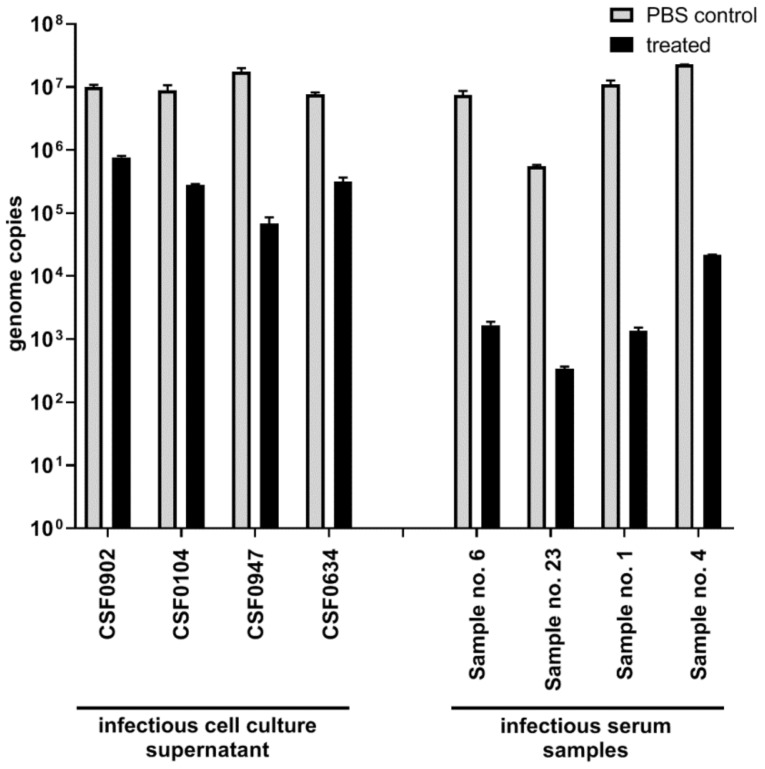
Classical swine fever virus (CSFV) genome detection by quantitative real-time RT-PCR (qRT-PCR) after sample treatment for inactivation of CSFV. Four infectious cell culture supernatants and four infectious serum samples were 1:2 diluted in phosphate buffered saline (PBS)-Tween_20_ (PBS 0.05% Tween_20_ for cell culture supernatant and PBS 0.3% Tween_20_ for serum) and incubated at 56 °C for 30 min (treated). As a control for viral genome load, samples were 1:2 diluted in PBS and not incubated at 56 °C for 30 min (PBS control). All samples were analyzed in duplicates in a CSFV-specific qRT-PCR and the mean values are shown.

In the initial stage of the study, CSFV infectious cell culture supernatants of four different CSFV strains were used. The infectious cell culture supernatants showed a virus load between 1.78 × 10^6^ to 3.16 × 10^6^ tissue culture infection dose 50 (TCID_50_)/ 50 µL after 1:2 dilution in PBS (PBS control). Incubation at 56 °C for 30 min resulted in a decrease of the virus titer by approximately two log units [PBS + heat (H) control]. If the infectious supernatants were diluted in PBS containing 0.05% Tween_20_ (final concentration of Tween_20_: 0.025%) and heat treatment was performed, no infectious virus was detected (Figure 1A). This was confirmed by virus isolation on two different cell lines. The samples that were diluted in PBS without heat treatment (PBS control) were tested positive by virus isolation. Generally, detection of CSFV genome was still possible, but the samples that were treated with PBS 0.05% Tween_20_ showed a considerable lower number of genome copies compared to the samples treated with PBS (PBS control) (Figure 2).

However, after dilution of serum samples in PBS 0.05% Tween_20_ followed by heat treatment, infectious CSFV was still detectable. Accordingly, this inactivation protocol was not effective for serum samples, even though the initial virus titers in the four serum samples (ID 1, ID 4, ID 6, and ID 23) were significantly lower (2.14 × 10^2^ to 3.77 × 10^4^ TCID_50_/ 50 µL) than the virus titers in the infectious cell culture supernatants. The calculated virus titers of the serum samples after dilution in PBS 0.05% Tween_20_ were comparable to the titers of the serum samples that were diluted in PBS without Tween_20_ (PBS + H control). Likewise, dilution of the serum in PBS 0.1% Tween_20_ (final concentration of Tween_20_: 0.05%) followed by heat treatment showed no significant differences in the decrease of the virus titer compared to the PBS control that was incubated at 56 °C (PBS + H control). Only after dilution in PBS 0.3% Tween_20_ (final concentration of Tween_20_: 0.15%) combined with heat treatment was the infectious virus no longer detectable (Figure 1B), which was confirmed by virus isolation on two porcine cell lines. In comparison to this, all samples that were diluted in PBS without heat treatment (PBS control) were detected positive for infectious CSFV by virus isolation. As it was shown for the infectious cell culture supernatants, CSFV genome was still detectable after treatment with Tween_20_. However, the number of genome copies was reduced compared to the serum samples treated with PBS (PBS control) (Figure 2). The concentration of 0.3% Tween_20_ had no influence on cell growth, whereas the samples diluted in PBS 1% Tween_20_ or PBS 2% Tween_20_ induced a cytopathic effect. This cytopathic effect was observed within the first two dilution steps of virus titration and did not occur after incubation of the cells with higher dilutions of the PBS 1% Tween_20_ and PBS 2% Tween_20_-treated samples (data not shown). 

### 2.2. Validation of CSFV Inactivation 

To confirm the efficacy of the protocol for virus inactivation in serum samples and to evaluate the application of these treated samples in serological diagnostic assays [virus neutralization test (VNT) and ELISA], a panel of 33 sera was tested. The panel comprised two different sample categories. Samples of category I were tested positive for infectious CSFV whereas samples of category II were classified negative for infectious CSFV. 

The serum samples were 1:2 diluted in PBS 0.3% Tween_20_ or PBS 0.5% Tween_20_ and incubated at 56 °C for 30 min. Immediately after treatment, CSFV positive sera of category I (*n* = 23) treated with PBS 0.3% Tween_20_ were subjected to virus isolation over two passages using the two porcine cell lines, porcine kidney (PK15) and swine kidney (SK6), separately. None of the 23 treated serum samples tested positive for infectious virus, neither in the undiluted approach nor in the 1:10 dilution. The positive control clearly tested positive for infectious virus confirming that the method has been performed properly. For the detection of CSFV-specific antibodies within a short time, commercial ELISAs are often used. In case a serum sample is tested positive or doubtful by the ELISA, an additional confirmatory test is performed, generally the VNT. 

**Figure 3:**
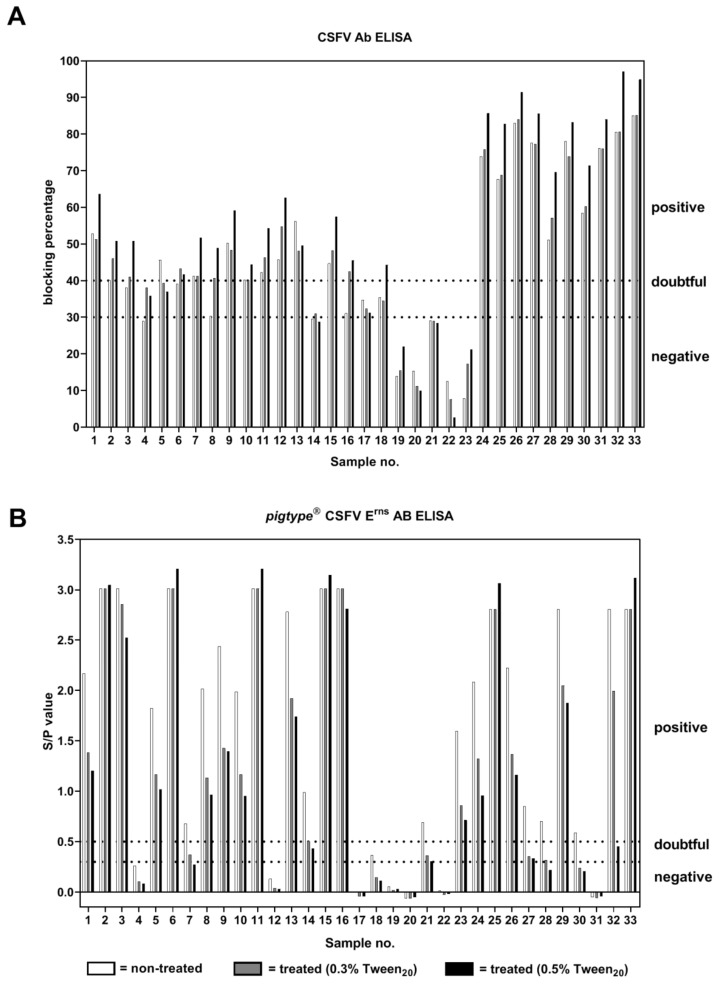
Detection of antibodies against Classical swine fever virus by two ELISAs. A total of 33 serum samples were treated with phosphate buffered saline (PBS) 0.3% Tween_20_ or PBS 0.5% Tween_20_ and incubated at 56 °C for 30 min. Afterwards, these samples were analyzed in comparison to non-treated samples in an E2-specific (**A**) and E^rns^-specific (**B**) ELISA.

Previous studies have described differences in the detection of antibodies using ELISAs that are based on either E2 or E^rns^ antigen [11]. Against this background, the samples of the present study were tested in an E2-specific (CSFV Ab ELISA; IDEXX) as well as in an E^rns^-specific ELISA (*pigtype^®^* CSF E^rns^ AB ELISA; Indical). The results of the E2-specific ELISA showed no significant differences in the detection of antibodies between treated and non-treated samples. Also, the classification “positive for antibodies against CSFV” of samples was not negatively influenced by the treatment with PBS 0.3% Tween_20_ or PBS 0.5% Tween_20_, except for one sample (sample ID 5). However, the samples that were treated with PBS 0.5% Tween_20_ showed rather higher values of blocking percentage. Four non-treated samples (ID 3, ID 6, ID 8, and ID 16) were tested doubtful for antibodies against CSFV, but positive for antibodies against CSFV after treatment with Tween_20_ (0.3% or 0.5%) and incubation at 56 °C, and one negative tested non-treated sample (ID 4) showed a doubtful result after treatment (Figure 3A). 

The results obtained by the E^rns^ antibody ELISA were different compared to the detection of antibodies against E2 (Figure 3B). One major difference was that high amounts of E^rns^-specific antibodies were detected in the majority of the serum samples of category I (sample IDs 1–16 and ID 23). However, several samples that were treated with Tween_20_ and heat showed decreased S/P values (ratio of sample OD_450_ to mean OD_450_ of the positive control) compared to the non-treated samples (S/P value difference ≤ 1). This had an impact on the classification of samples with a low antibody response. As a result, five treated samples (ID 7, ID 21, ID 27, ID 28, and ID 30) were classified as doubtful or negative for antibodies against CSFV instead of a positive result obtained by analysis of untreated samples. Sample 14 tested positive, with an S/P value very close to the cut-off (S/P value = 0.504) after treatment with PBS 0.3% Tween_20_. This sample was classified doubtful (S/P value = 0.459) after treatment with PBS 0.5% Tween_20_. However, this inter-test variance is not significant and generally observed for repetition of ELISAs. Only for one sample (ID 32) was a high difference in S/P value after treatment with PBS 0.3% Tween_20_ or PBS 0.5% Tween_20_ detected. Generally, the S/P values, which were obtained for the samples treated with PBS 0.5% Tween_20_ were slightly lower than the ones detected after treatment with 0.3% Tween_20_. These minor differences did not lead to additional difference in sample classification, except for one sample (ID 32). 

**Figure 4:**
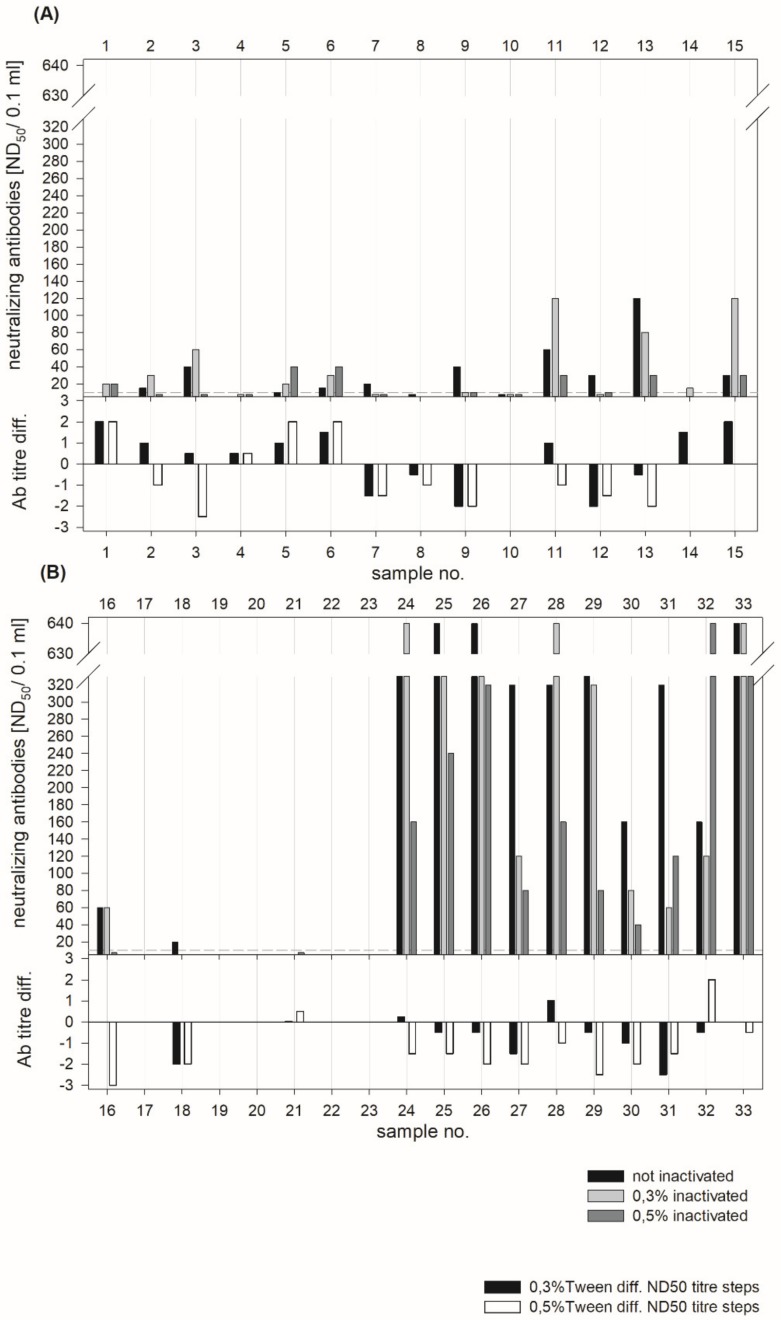
Detection of neutralizing antibodies against Classical swine fever virus by virus neutralization test (VNT) after sample treatment. A total of 33 serum samples were treated with phosphate buffered saline (PBS) 0.3% Tween_20_ or PBS 0.5% Tween_20_ and incubated at 56 °C for 30 min. Afterwards, these samples were analyzed in comparison to non-treated samples in the VNT. In addition to the titers of neutralizing antibodies, titer differences between the treated and non-treated samples are depicted. A negative value for the titer difference showed a reduction of the antibody titer compared to the non-treated samples. Results for samples ID 1–15 are summarized in (**A**) and those for the sample ID 16–33 in (**B**).

As the VNT is often used for confirmation of positive or doubtful ELISA results, as well as for the differentiation between CSFV-specific and ruminant pestivirus-specific antibodies, the 33 serum samples were also tested in the VNT. The detection of virus neutralizing antibodies was performed immediately after dilution in PBS 0.3% Tween_20_ or PBS 0.5% Tween_20_ along with heat treatment. No negative effects caused by the Tween_20_ concentrations were detected. A low-to-moderate neutralizing antibody titer (up to 120 ND_50_) was detected for the samples of category I (sample ID 1–23). Sera of category II (sample ID 24–33) showed a moderate-to-high neutralizing antibody titer (>640 ND_50_). For most treated samples, a slight increase of the antibody titer was observed compared to the non-treated samples. However, a difference of up to three titer steps is commonly detected by repetition of VNTs and is therefore considered not significant. A threefold or higher difference in the antibody titer detected for treated and non-treated samples was not observed, except for one sample (ID 16) after treatment with PBS 0.5% Tween_20 _(Figure 4). 

On the basis of the fact that no significant titer differences were observed for treated and non-treated samples, it can be concluded that the concentration of Tween_20_ (final concentration after 1:5 dilution in the VNT: 0.03% Tween_20_ or 0.05% Tween_20_) had no significant influence on inactivation of the CSFV strain CSF0104 (100 TCID_50_) that was used for performing the VNT. 

According to the results, 11 out of the 33 serum samples were apparently hemolytic. However, this characteristic had no influence on serological analysis in VNT and ELISA using treated samples.

## 3. Discussion

A rapid and safe detection of notifiable epizootic diseases, such as CSF, is of great importance. To keep the diagnostic methods up to date and to ensure a continuous efficient diagnostic performance, a regular exchange of reference material is indispensable. Therefore, reference material is often requested for verification and validation of diagnostic assays applied for detection of CSFV genomes or antibodies against CSFV. Usually, this sample material is obtained from animals that were infected with CSFV. Despite of appropriate testing [e.g., by quantitative real-time RT-PCR (qRT-PCR) or virus isolation] a contamination or residual virus in samples that were taken from animals infected with CSFV cannot be ruled out in general. For this reason, minimization of the risk of any viable CSFV in serum samples is of utmost importance, especially when the sample material is intended for laboratories that are not allowed to work with infectious viruses or that have a strict separation between serological and virological working departments. The significance of the CSFV inactivation is underlined by the fact that this topic had been already targeted before. However, former studies exclusively addressed virus inactivation in tissues or other matrices that are not relevant for serological analysis upon shipping [5,12,13]. The focus of the present study was to define a pragmatic procedure for inactivation of CSFV in serum without impairing the serological properties for subsequent antibody detection by ELISA and cell culture-based VNTs. 

It has been shown for other enveloped viruses (Ebola virus and Rift Valley fever virus) that virus inactivation in serum samples can be performed by addition of the non-ionic detergent Tween_20_ (final concentration: 0.5%) and incubation at 56 °C for 1 h [6,7,8]. Tween_20_ solubilizes lipid membranes and is widely used as a component of washing buffers to prevent unspecific binding of antibodies, for example, in ELISAs and immune blots. The former studies showed that serum samples, which were treated with Tween_20_ and were incubated at 56 °C for 1 h, are suitable for antibody detection by ELISAs. However, the application in other serological assays involving cell culture systems, for example, VNT, has not been evaluated so far. VNT is one of the most important serological assays for the detection of antibodies against CSFV, particularly as a confirmatory test for positive or doubtful ELISA results, and for the differential diagnosis regarding antibodies against other closely related pestiviruses [10]. The present study showed that the use of 1% Tween_20_ (final concentration: 0.5%) led to virus inactivation in serum samples but cytotoxic effects were detected in cell culture. These effects can be traced back to the high Tween_20_ concentration. On the basis of these results, it can be concluded that samples treated with PBS 1% Tween_20_ cannot be used for analysis in cell culture-based techniques such as VNT. In contrast, no cytotoxic effects were observed for lower Tween_20_ concentrations (≤0.5% Tween_20_ in PBS).

Fortunately, virus inactivation was also confirmed for serum samples that were treated with a lower concentration of Tween_20_ (PBS 0.3% Tween_20_ or PBS 0.5% Tween_20_) without impairing the growth of tissue culture cell. Furthermore, the present study showed that serum samples, which were inactivated using PBS 0.3% Tween_20_ (final concentration: 0.15%) or PBS 0.5% Tween_20_ (final concentration: 0.25%) and heat treatment (56 °C for 30 min), can be used for serological assays to detect antibodies against CSFV by ELISA and VNT. One limitation of the inactivation protocol was observed for the *pigtype^®^* CSFV E^rns^ AB ELISA that includes a 1:10 dilution of the sample prior to analysis. Five inactivated serum samples that were treated with PBS 0.3% Tween_20_ (ID 7, ID 21, ID 27, ID 28, and ID 30) were classified doubtful or negative for antibodies against CSFV, whereas the corresponding non-treated samples were tested positive. However, this finding may be tolerable because the majority of routinely applied serological assays target either E2-specific antibodies or the NS3 of CSFV (common staining method of VNT). It was shown that a dilution factor of two, which was used for the CSFV E2-specific AB ELISA and the VNT, had no significant influence on sample classification. Moreover, the present study confirmed that the immune response against the structural proteins E^rns^ and E2 is different [11]. A high amount of antibodies against E^rns^ was detected at an early time point after infection or in serum samples that contain virus. In comparison to this, all samples that do not contain infectious CSFV showed highly positive results in the E2-specific antibody ELISA. For the analysis of CSFV genome by quantitative real-time RT-PCR, the inactivation method is not recommended because addition of Tween_20_ to the sample had a negative impact on detection of viral genomic RNA. However, the implementation of this inactivation technique was not focused on subsequent CSFV genome detection as this is considered not relevant for serological departments that are forbidden to work with infectious viruses. 

Interestingly, inactivation of CSFV was dependent on the matrix. For inactivation of CSFV in cell-free supernatant, the required Tween_20_ concentration was considerably lower (final Tween_20_ concentration: 0.025%) than for CSFV inactivation in serum samples (final Tween_20_ concentration: 0.15%), even though the virus titers in serum samples (2.14 × 10^2^ to 3.77 × 10^4^ TCID_50_/ 50 µL) were about 100- to 10,000-fold lower than the virus titers in tissue culture supernatants (1.78 × 10^6^ to 3.16 × 10^6^ TCID_50_/ 50 µL). A similar concentration of Tween_20_ was also used for inactivation of West Nile virus in cell-free supernatant [9]. It can be hypothesized that the presence of proteins in serum samples may influence the efficiency of inactivation by detergent Tween_20_ in lower concentrations. In this context, it has been reported that, even though CSFV can be considered as a moderately fragile virus, prolonged survival is possible under favorable conditions such as a protein-rich milieu [13]. Different results have been published with regard to the influence of the properties of the viral strain on virus stability [5,12,14]. In the present study, no differences regarding virus inactivation were detected between the highly virulent strains CSF0947 and CSF0634 and the two moderately virulent strains CSF0902 and CSF0104. 

In order to prove a broad application of the inactivation technique, a serum panel has been compiled using the serum collection of the EU and OIE Reference Laboratory for CSF (Hannover, Germany). This validation panel comprised sera with different properties, for example, samples that were taken from animals infected with distinct CSFV genotypes, as well as samples containing various antibody and genome amounts. The virus titers of the serum samples used in this study are representative of those typically found after CSFV infection. The EU and OIE Reference Laboratory for CSF organizes an international inter-laboratory comparison test every year to evaluate the diagnostic methods including virus isolation and virus titration. The titer of these sera, which were taken from experimentally infected pigs, ranges between 10^1^ and 10^5.5^ TCID_50_/50 µL. 

Each of the virus positive samples (*n* = 23) were completely inactivated by the suggested method. Due to the fact that virus isolation on CSFV-susceptible cells was performed immediately after sample treatment, it can be concluded that the virus inactivation resulted from the treatment itself, instead of any other possible influence of subsequent storage of the sample in PBS-Tween_20_. However, the volume of serum samples to be inactivated needs to be considered for the incubation time at 56 °C because the time for reaching the core temperature of 56 °C within the serum sample depends on the sample volume. Accordingly, for sample volumes > 2 ml, longer incubation times are recommended. 

The present study shows that treatment with PBS-Tween_20 _(0.5%) and incubation at 56 °C for 30 min leads to complete virus inactivation. For laboratories that are not allowed to work with infectious CSFV, safety precautions must be applied before these laboratories can receive sample material that was taken from CSFV-infected animals. So far, only samples that were tested negative for CSFV genome and for infectious CSFV are shipped. As an additional safety measure, the laboratories are informed (e.g., by the corresponding material transfer agreement) that adequate disinfection and decontamination of all things that came into contact with the material are requested. The present study showed that treatment with PBS-Tween_20_ combined with complement inactivation represents an additional third safety margin for serum samples, which are tested negative for CSFV genome beforehand. Furthermore, this treatment represents a good alternative for laboratories that have a separation between serology (non-infectious) and virology departments. For these laboratories, the described method for CSFV inactivation offers the possibility to analyze individual serum samples first by virological methods, followed by serological analysis after sample treatment with PBS-Tween_20_ and heat. However, because the inactivation of an agent causing a notifiable disease is a serious issue, it is recommended to confirm virus inactivation of any sample beforehand.

In conclusion, in order to minimize the risk of CSFV contamination at the consignee’s premises regarding laboratories that have no permission for working with infectious CSFV, the treatment of CSFV genome negative serum samples with Tween_20_ (final concentration should be 0.15%) and incubation at 56 °C for 30 min prior to shipment can be recommended. It is a simple, pragmatic, and cost-efficient approach for CSFV inactivation that can be performed by each laboratory as it does not require any special equipment. However, this method needs to be carefully validated in each laboratory beforehand and longer incubation times should be applied for samples containing volumes >2 ml.

## 4. Materials and Methods

### 4.1. Serum Samples

In total, 33 serum samples that were taken from animals experimentally infected with CSFV were used in the present study. These samples were allocated into two different categories. Samples of category I tested positive for infectious CSFV and developed no or a low-to-moderate immune response against CSFV (*n* = 23; ID 1 to ID 23). Furthermore, 9 out of the 23 samples were visibly hemolytic (ID 2, ID 4, ID 5, ID 6, ID 9, ID 13, ID 16, ID 20, and ID 23). Category II comprised sera tested negative for infectious CSFV, but clearly positive for CSFV antibodies (*n* = 10; ID 24 to ID 33). Two of these samples were hemolytic (ID 27 and ID 31). All samples were obtained from the serum sample collection of the EU and OIE Reference Laboratory for CSF (Hannover, Germany). The corresponding animal experiments were notified by the Specialized Department of Animal Welfare Service of the Lower Saxony State Office for Consumer Protection and Food Safety (LAVES; Permit Number: LAVES AZ 08A 538) according to the German animal welfare act.

### 4.2. Cell Culture and Viruses

Two different cell lines, PK15 and SK6 cell line, were used in the present study. The cell line PK15 (CCLV0051) was obtained from the Collection of Cell Lines in Veterinary Medicine (CCLV, Friedrich-Loeffler-Institute, Island of Riems, Greifswald, Germany) and was cultivated in Eagle’s minimum essential medium (EMEM), supplemented with fetal calf serum (FCS); 5% (for SK6 cells) or 7.5% (for PK15 cells) FCS were added to the growth medium and 10% to the virus culture medium. 

SK6 cells were kindly provided by the Institute of Virology and Immunoprophylaxis, Mittelhäusern, Switzerland, and were grown in Dulbecco’s modified Eagle’s medium supplemented with 10% FCS. Before usage, FCS was tested for absence of pestivirus genomes and pestivirus-specific antibodies.

Two highly (CSF0947 and CSF0634) and two moderately (CSF0902 and CSF0104) virulent CSFV strains were applied for validation of virus inactivation. These virus strains derive from the virus collection at the EU and OIE Reference Laboratory for CSF in Hannover, Germany [15].

### 4.3. Inactivation of CSFV by Treatment with Tween_20_ and Heat 

The first inactivation experiment was performed using infectious cell culture supernatants with high viral loads (TCID_50_ > 10^6^/50 µL), two highly (CSF0947 and CSF0634) and two moderately virulent strains (CSF0902 and CSF0104), independently. Infectious cell culture supernatants were 1:2 diluted in PBS containing 0.05% Tween_20_, which represents a commonly used Tween_20_ concentration in blocking reagents and washing buffers of antibody ELISA kits. The diluted samples were incubated at 56 °C for 30 min in a water bath. Immediately after heat treatment, virus infectivity was quantified by sample titration on CSFV-susceptible cells according to the EU Diagnostic Manual for CSF Diagnosis, Technical Part [16], in three independent runs, and virus isolation as well as qRT-PCR were performed. Two PBS controls were included—for the first, the samples were 1:2 diluted in PBS and incubated at 56 °C for 30 min (PBS + H control), whereas the second control was not treated with heat (PBS control).

Additionally, CSFV inactivation in serum samples was investigated. Four serum samples containing infectious CSFV (2.14 × 10^2^ to 3.77 × 10^4^ TCID_50_/ 50 µL) were 1:2 diluted in PBS supplemented with different Tween_20_ concentrations (0.05%, 0.1%, 0.3%, 0.5%, 1%, and 2%) and incubated at 56 °C for 30 min in a water bath. As described above, two PBS controls were included and the samples and were analyzed by virus titration, virus isolation, and qRT-PCR, as described above.

### 4.4. Validation of the Inactivation Protocol

The protocol for inactivation was validated using a panel consisting of 33 serum samples with different virological and serological properties. To confirm virus inactivation, the samples were treated with PBS 0.3% Tween_20_ and heat (56 °C for 30 min), and thereafter subjected to virus isolation. Subsequently, serological analysis (VNT, CSFV Ab ELISA (IDEXX) and *pigtype^®^* CSF E^rns^ AB ELISA (Indicial)) were performed. To consider a safety margin regarding virus inactivation, the samples were analyzed after treatment with PBS 0.5% Tween_20_ and also heat. For comparison, untreated serum samples were also analyzed. 

### 4.5. Virus Isolation

Virus isolation was performed using the following three sample set-ups: (1) four infectious cell culture supernatants treated with heat and PBS 0.05% Tween_20_ or PBS (PBS control), (2) four serum samples treated with heat and PBS 0.3% Tween_20_ or PBS (PBS control), and (3) serum samples of category I out of the serum validation panel that were diluted in PBS 0.3% Tween_20_ and incubated for 30 min at 56 °C. The protocol for virus isolation followed the instructions described in the EU Diagnostic Manual for CSF Diagnosis, Technical Part [16]. Briefly, two concentrations of the serum samples (undiluted and 1:10 diluted) were incubated on PK15 and SK6 cells for three days, respectively. A positive and negative control was treated equally to the sample material. After two passages, cell culture supernatant was removed and the cells were fixed by heat treatment (80 °C for 3 h). Subsequently, an indirect immune-peroxidase staining was performed using the NS3-specific monoclonal mouse antibody BVD/C16 (dilution 1:25 in PBS-0.01%Tween_20_) and a polyclonal rabbit anti-mouse horseradish peroxidase conjugate (dilution 1:200 in PBS-0.01% Tween_20_, DAKO, Denmark).

### 4.6. ELISAs for Detection of Antibodies Against CSFV

For antibody detection against CSFV, two commercially available ELISAs were carried out according to the manufactures instructions. The antibody ELISAs are different with regard to antigen specificity, the CSFV Ab ELISA (IDEXX) detects antibodies against the structural glycoprotein E2 and the *pigtype^®^* CSFV E^rns^ AB ELISA (Indicial) against the structural protein E^rns^. 

### 4.7. Virus Neutralization Assay

The VNT has been performed according to the standard protocol described in the EU Diagnostic Manual for CSF Diagnosis, Technical Part [16], starting with a 1:5 dilution of the serum sample. The final applied dilution was 1:640. The cell line PK15 and the CSFV reference strain Diepholz (CSF0104; genotype 2.3) were used. 

### 4.8. Isolation of Viral RNA and qRT-PCR

To analyze the influence of Tween_20_ on genome detection by qRT-PCR, total RNA was extracted from four serum samples and four infectious cell culture supernatants that were diluted in PBS 0.3% Tween_20_ along with incubation at 56 °C for 30 min or PBS without heat treatment (PBS control), independently. For this purpose, the QIAamp Viral RNA Mini Kit (Qiagen, Germany) was used according to the manufacturers’ recommendations. Afterwards, viral RNA was detected by a CSFV-specific qRT-PCR, as described previously [17]. The samples were analyzed in duplicate.
